# Double-ligand modulation for engineering magnetic nanoclusters

**DOI:** 10.1186/1556-276X-8-104

**Published:** 2013-02-22

**Authors:** Bongjune Kim, Jaemoon Yang, Eun-Kyung Lim, Joseph Park, Jin-Suck Suh, Hyo Seon Park, Yong-Min Huh, Seungjoo Haam

**Affiliations:** 1 Department of Chemical and Biomolecular Engineering, College of Engineering, Yonsei University, Seoul, 120-749, Republic of Korea; 2 Department of Radiology, College of Medicine, Yonsei University, Seoul, 120-752, Republic of Korea; 3 Department of Architectural Engineering, College of Engineering, Yonsei University, Seoul, 120-749, Republic of Korea

**Keywords:** Magnetic nanoparticle, Double-ligand modulation, Nanoemulsion, Magnetic nanocluster, Magnetic resonance imaging

## Abstract

Magnetic nanoclusters (MNCs) are agglomerated individual magnetic nanoparticles (MNPs) that show great promise in increasing magnetic resonance imaging (MRI) sensitivity. Here, we report an effective strategy to engineer MNCs based on double-ligand modulation to enhance MRI sensitivity. The oleic acid-coated individual MNPs self-assembled and then were enveloped by polysorbate 80, using a nanoemulsion method to prepare MNCs. By modulating the amounts of the two ligands, and thus the size and magnetic content of the resultant MNCs, we were able to enormously improve MRI sensitivity.

## Background

Magnetic resonance imaging (MRI) is a powerful imaging tool for clinical diagnosis due to noninvasive tomographic imaging potentials with high spatial resolution [1-5]. In particular, MRI using magnetic nanoparticles (MNPs) conjugated to a targeting moiety is a highly attractive approach for the molecular imaging of cancer-specific biomarkers. This is because the T2-shortening effect of MNPs results in dark contrast [5-13]. Studies aimed at increasing T2 MRI sensitivity report that increasing the magnetization value by size growth and metal doping enhances the T2 shortening effect [8-10]. However, the size increase induced the superparamagnetic-ferromagnetic transition, so resulting MNPs were no longer suitable as MRI contrast agents.

Recent efforts in nanocrystal synthesis have shifted to secondary structure manipulation to upgrade the properties of individual nanocrystals based on interactions between their subunits [14-18]. Magnetic nanoclusters (MNCs) as a secondary structure are composed of assembled MNPs that reportedly can act as contrast agents to improve T2 MRI capability. Precisely, MNCs showed higher T2 relaxivity and a larger darkening effect than individual MNPs because they possess higher magnetization per particle with superparamagnetic property [19-24]. MNCs have been fabricated either by self-assembly or through direct solution growth. The common goal of these synthetic methods was to control the size of MNCs because T2 relaxivity increases are proportional to particle size [23,24]. However, the signal enhancement provided by MNCs still remains unsatisfactory because the studies about the density of individual MNPs consisting MNCs have not been concerned yet. Thus, a primary issue in MNC fabrication is to optimally increase magnetic content in concert with particle enlargement to improve T2 relaxivity.

**Figure 1:**
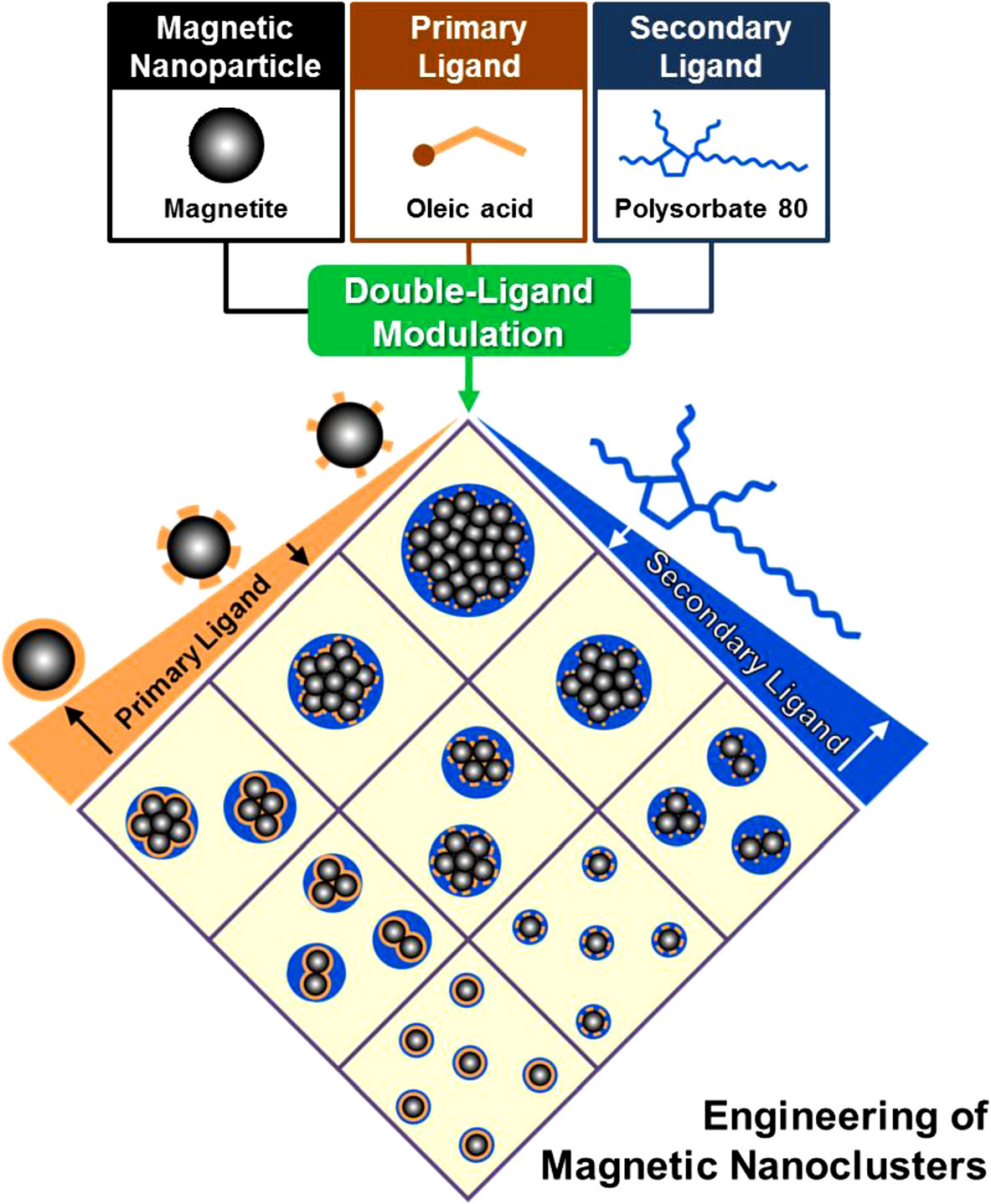
Schematic illustration for engineering MNCs based on double-ligand modulation.

Herein, we developed an effective strategy to selectively engineer MNC particle size and magnetic content, using a double-ligand modulation approach, to enhance T2 MRI signal intensity. First, high-quality MNPs exhibiting strong nanomagnetism were synthesized by thermal decomposition. High-quality MNPs composed MNCs to derive effective enhancement of MNC T2 relaxivity. Second, a series of MNPs possessing various weight percent of oleic acid (primary ligand) was prepared. This allowed us to control MNP-MNP distances when these particles were combined to create MNC agglomerates, thereby regulating MNC density to our desired specifications. Finally, primary ligand-modulated MNPs were assembled and encapsulated using polysorbate 80 (secondary ligand) by nanoemulsion to construct MNCs. During nanoemulsion, various MNC sizes were fabricated by manipulating the concentration of polysorbate 80 employed. Subsequently, we compared the T2 relaxivity of these different MNCs to assess the optimal synthesis conditions for achieving optimal T2 signal enhancement. A conceptual scheme of the double-ligand modulation strategy for engineering MNCs is shown in Figure 1.

## Methods

### Materials

Iron(III) chloride hexahydrate, sodium oleate, oleic acid, 1-octadecene, and polysorbate 80 (polyoxyethylene sorbitan mono-oleate) were purchased from Sigma-Aldrich Co. (St. Louis, MO, USA). All other chemicals and reagents were of analytical grade.

### Synthesis of iron-oleate complex

Iron-oleate complex was prepared by reacting iron chloride and sodium oleate. For the synthesis, 10.8 g (40 mmol) iron chloride and 36.5 g (120 mmol) sodium oleate were dissolved in a mixed solvent composed of 80 mL ethanol, 60 mL deionized water, and 140 mL *n*-hexane. The resulting solution was heated to 70°C for 4 h. When the reaction was completed, the upper organic layer containing the iron-oleate complex was washed three times with 30 mL deionized water, using a separation funnel. After washing, residual *n*-hexane was evaporated off, leaving the iron-oleate complex as a waxy solid [25].

### Synthesis of iron oxide MNPs

Thirty-six grams (40 mmol) of the synthesized iron-oleate complex and 5.7 g (20 mmol) oleic acid were dissolved in 200 g 1-octadecene at room temperature. The resulting solution was heated to 320°C with a constant heating rate of 3.3°C min^−1^, and then reacted at 320°C for 30 min. The resulting solution containing the MNPs was cooled to room temperature, and 500 mL ethanol was added to the solution. The MNPs were purified by centrifugation and resuspended in *n*-hexane [25,26].

### Preparation of primary ligand-modulated MNPs containing various amounts of oleic acid (primary-ligand modulation)

Excess oleic acid was removed from synthesized MNPs by ethanol precipitation. Fifty milliliters of ethanol was added to the 50 mg MNPs dissolved in 5 mL *n*-hexane, and the resulting mixture was sonicated at 190 W for 20 min. After sonication, MNPs were separated by centrifugation (99×*g*, 10 min) and resuspended in 5 mL *n*-hexane. After ethanol precipitation, primary ligand-modulated MNPs (PMNPs) containing the lowest amount of oleic acid (LMNPs) were obtained, and the other PMNPs containing medium (MMNPs) and the highest (HMNPs) amount of oleic acid were prepared by adding pure oleic acid to the LMNPs.

### Preparation of MNCs by the nanoemulsion method (secondary-ligand modulation)

Four milliliters of *n*-hexane containing 10 mg LMNPs was added to 20 mL deionized water containing 100, 50, 25, or 10 mg polysorbate 80. After mutual saturation of the organic and aqueous phases, the mixture was sonicated for 20 min at 190 W with vigorous stirring. After sonication, the organic solvent was evaporated rapidly using a rotary evaporator to form MNCs. The other PMNPs (MMNPs and HMNPs) were similarly used to prepare MNCs by the identical nanoemulsion method [27].

### Magnetic resonance imaging

Magnetic resonance imaging experiments were performed with a 1.5-T clinical MRI instrument with a Micro-47 surface coil (Intera, Philips Medical Systems, Amsterdam, The Netherlands). T2 relaxivity (*r*2 (s^−1^ mM^−1^); ratio of R2 (1/T2) to iron concentration) of MNCs was measured at room temperature by the Carr-Purcell-Meiboom-Gill sequence: TR = 10 s, 32 echoes, with 12 ms even echo space, number of acquisitions = 1, point resolution 156 × 156 μm, section thickness 0.6 mm.

### Characterization

The morphology and the size of MNPs were analyzed using a transmission electron microscope (JEM-2100 LAB6, JEOL Ltd., Akishima-shi, Japan), and the crystallographic structure of MNPs was obtained from X-ray diffraction patterns (D/MAX Ultima III, Rigaku Co., Shibuya-ku, Japan). The characteristic bands of pure oleic acid and MNPs were evaluated by Fourier transform infrared spectroscopy (FT-IR; Excalibur Series, Varian Inc., Palo Alto, CA, USA) to confirm the existence of oleic acid on the MNPs. The amount of oleic acid on the MNPs was quantified using a thermogravimetric analyzer (SDT-Q600, TA Instruments, New Castle, DE, USA). The MNC size (hydrodynamic diameter) was analyzed by laser scattering (ELS-Z, Otsuka Electronics, Hirakata-shi, Japan). The Fe concentration in MNCs was quantified by inductively coupled plasma atomic emission spectrometry (Thermo Electron Corporation, Waltham, MA, USA).

## Results and discussion

**Figure 2:**
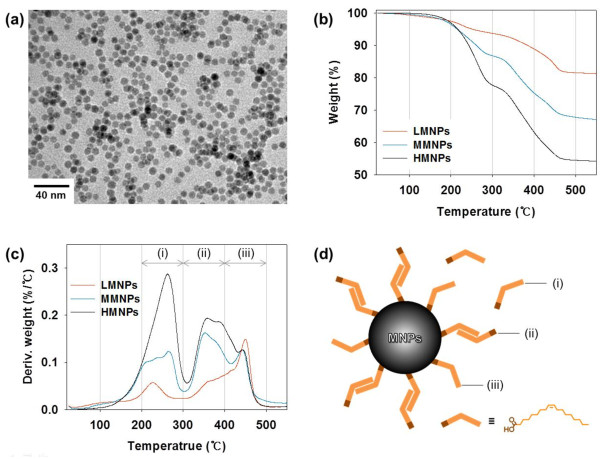
**Characterization of PMNPs.** (**a**) Transmission electron microscopy image of MNPs. (**b**) Thermogravimetric analysis shows weight change in relation to temperature of the three PMNPs containing different amounts of primary ligand (oleic acid). (**c**) Derivative weight curves of the three PMNPs (LMNPs, MMNPs, and HMNPs). (**d**) Illustration of the interactions of oleic acid on MNPs.

High-quality MNPs in terms of size uniformity, single crystallinity, and high magnetism should be verified first as a part of the building blocks that comprise the MNCs. This guarantees repeatability in experiments aimed to determine optimal enhancement of MNC T2 relaxivity. For particle uniformity, MNPs were synthesized by a thermal decomposition method using an iron-oleate as the precursor and oleic acid as the primary ligand [25]. The narrow size distribution (7.8 ± 0.5 nm) and the spherical morphology of the MNPs were ascertained by transmission electron microscopy (Figure 2a). The highly crystalline MNP structure was confirmed by the X-ray powder diffraction pattern assigned at 2*θ* values of 30° (220), 36° (311), 44° (400), 58° (511), and 63° (440), which indicated the inverse spinel structure of magnetite (Fe_3_O_4_; JCPDS no. 19–0629; Additional file 1: Figure S1a). Moreover, the MNPs exhibited the saturation magnetization value of 87 emu g^−1^ Fe at 1.0 T without magnetic hysteresis (Additional file 1: Figure S1b).

To control MNP clustering synthesis, the amount of oleic acid on the synthesized MNPs should be first modulated. This is necessary because the amount of oleic acid affects MNC formation. Steric repulsion among the hydrocarbon tails of oleic acid on individual MNPs impacts assembly capability of individual MNPs. To modify the amount of oleic acid on the MNPs, the MNPs were dissolved in *n*-hexane and ethanol was added to the solution to remove part of the oleic acid coating. Finally, three samples of PMNPs were successfully obtained from the precipitates [25,26], each coated with different oleic acid amounts: 19 (low PMNPs, LMNPs), 33 (medium PMNPs, MMNPs), and 46 (high PMNPs, HMNPs) wt.% (Figure 2b).

To investigate the effect of primary ligand on MNCs, the interactions of oleic acid molecules on the surface of MNPs were analyzed through derivative weight curves of the three samples of PMNPs (Figure 2c). These PMNPs showed three derivative peaks positioned between 25°C and 550°C [28-30]. The first peak positioned at approximately 250°C (Figure 2c, i) was due to the removal of free oleic acid molecules surrounding the MNPs (Figure 2d, i), consistent with the derivative peak of pure oleic acid (Additional file 1: Figure S2). The second peak positioned at approximately 350°C (Figure 2c, ii), which was close to the boiling temperature of oleic acid, indicated bilayered oleic acid molecules with hydrophobic interactions between hydrocarbon tails (Figure 2d, ii). The third peak at approximately 450°C (Figure 2c, iii) corresponded to oleic acid molecules covalently bound to MNPs (Figure 2d, iii). The characteristic peaks of the oleic acid-MNP conjugates from asymmetric and symmetric COO^−^ stretches of oleic acid (1,630 and 1,532 cm^−1^) were confirmed by FT-IR spectroscopy (Additional file 1: Figure S3 and Table S1) and were categorized as a chelating bidentate complex: peak separation as 98 cm^−1^ = 1,630 to 1,532 cm^−1^ (Additional file 1: Table S2) [30,31]. The derivative weight curve of an iron-oleate precursor used for MNP synthesis also agreed with the derivative peaks of PMNPs (Additional file 1: Figure S4). From these results, it was determined that LMNPs contained mostly surface-bound oleic acid molecules showing a sharp peak approximately 450°C (Figure 2c, red line). Increased oleic acid in MMNPs formed a surface bilayer, which showed as an additional derivative peak at approximately 350°C (Figure 2c, blue line). The appearance of a sharp peak at approximately 250°C in HMNPs represented excess free oleic acid molecules (Figure 2c, black line). Therefore, we expected that (1) LMNPs were more likely to agglomerate and form large dense MNCs, (2) MMNPs would undergo less self-assembly and form smaller MNCs compared with LMNPs, and (3) excess free oleic acid in HMNPs would disrupt the assembly of individual MNPs to form MNCs.

Following primary-ligand modulation, PMNPs were then emulsified with the nanoemulsion method, using polysorbate 80 as a secondary ligand to fabricate MNCs. The nanoemulsion was created by dropwise injection of a PMNP-laden organic solvent phase into an aqueous continuous phase, followed by ultrasonication and vigorous stirring. The nanoemulsion surface was then stabilized using polysorbate 80 dissolved in an aqueous phase. The PMNPs within the nanoemulsion assembled and packed into MNCs during solvent evaporation [23,27,32]. To control MNC size for maximizing T2 relaxivity, the polysorbate 80 concentration was adjusted. Polysorbate 80 is a surfactant that decreases MNC size by reducing emulsion surface tension. Therefore, the three PMNP samples were each emulsified with various amounts of polysorbate 80 (10, 25, 50, or 100 mg; 24-mL total reaction volume).

**Figure 3:**
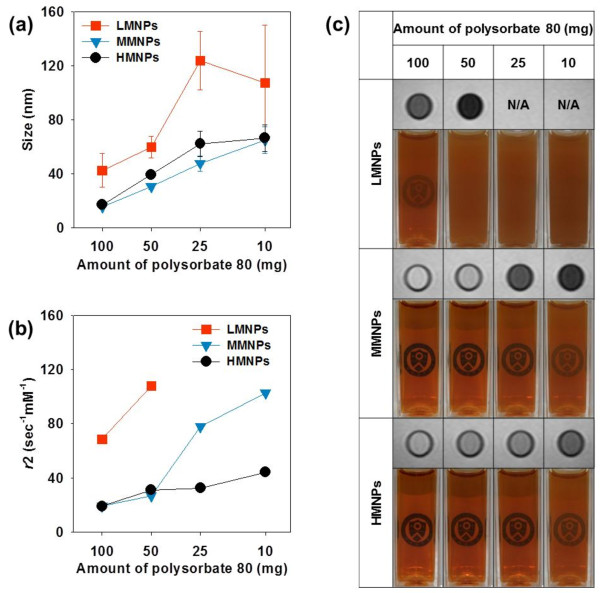
**Characterization of MNCs fabricated from three PMNPs.** (**a**) The size and (**b**) T2 relaxivity (*r*2) of MNCs. (**c**) Representative images of MNC solutions in the cubic cell and solution MRIs (0.74 mM Fe).

We compared the effect of varying oleic acid and polysorbate 80 concentrations on engineered MNC size, as determined by laser scattering. In Figure 3a, LMNPs formed larger MNCs at each polysorbate 80 concentration, than did the other two PMNPs. This is because LMNPs are coated with the least amount of oleic acid and thus possess the lowest level of steric repulsion between MNPs. This allows LMNPs to easily agglomerate to form the largest MNCs [33,34]. The increased oleic acid on MMNPs hindered the clustering of individual MNPs, resulting in smaller MNCs compared with LMNPs. The additional oleic acid molecules on HMNPs resulted in slightly bigger sized MNCs than MMNPs due to oily space occupied by excess oleic acid, at all polysorbate concentrations tested (detailed values for MNC sizes are presented in Additional file 1: Table S3). These results agreed with the observations of the derivative weight curves and demonstrated that primary-ligand (oleic acid) modulation of MNPs considerably affected final MNC size.

With all three PMNPs, increasing the polysorbate 80 concentration caused a decrease in final MNC size (Figure 3a). When polysorbate 80, a surfactant, was concentrated enough to cover large surface areas, MNP interfacial energy was sufficiently lowered to cause formation of smaller MNCs. By contrast, low polysorbate 80 concentrations insufficiently stabilized the entire MNP surface area and allowed nanoemulsion aggregation to form larger MNCs [23,35]. Thus, MNC size is easily regulated by modulating the amount of secondary ligand (polysorbate 80).

We then investigated the T2 relaxivity (*r*2) of variously sized MNCs created by double-ligand modulation, using a 1.5-T MRI instrument (Figure 3b). Magnetic nanoclusters fabricated from LMNPs exhibited a threefold higher *r*2 value compared to MNCs generated from MMNPs and HMNPs. This effect was due to the larger MNC size and greater density of these MNCs. Magnetic nanoclusters composed of MMNPs exhibited higher *r*2 values than MNCs created from HMNPs, when 10 and 25 mg polysorbate 80 were employed. The MNCs fabricated from MMNPs had less oily space and higher MNP content than MNCs generated from HMNPs. At 50 and 100 mg polysorbate 80, however, MNCs fabricated from MMNPs and HMNPs showed no noticeable distinction in *r*2 values. The difference of oleic acid content in these two PMNPs is insufficient to differentiate the size and magnetic content of MNCs when high concentrations of polysorbate 80 are employed in the reaction. At excess polysorbate 80 concentrations, polysorbate 80 stabilized the MNCs to form quite small ones.

The MNC *r*2 value variations observed when using a constant amount of polysorbate 80 were derived by primary-ligand modulation. Additionally, the increased *r*2 values in concert with decreased polysorbate 80 concentrations in the reaction were caused by MNC size increases due to the effect of secondary-ligand modulation [23]. Thus, these results demonstrate that modulation of both primary and secondary ligands is crucial for engineering MNCs to provide maximally enhanced MRI sensitivity. The *r*2 values of MNCs created from LMNPs using low amount of polysorbate 80 (10 and 25 mg) were not measurable because unstable MNCs were aggregated under an external magnetic field. Detailed MNC *r*2 values are presented in Additional file 1: Table S3.

Figure 3c shows photographs of MNCs dispersed in water and their T2-weighted solution MRIs. MNCs prepared from MMNPs and HMNPs were well dispersed in water without sedimentation, whereas LMNPs showed aggregation with larger cluster size that gradually settled over time. This indicates that insufficient polysorbate 80 concentrations were employed to form stable nanoclusters (Additional file 1: Figure S5). In addition, T2-weighted solution MRIs of MNCs obtained at the same iron concentration (0.74 Fe mM) showed darker images with decreased amount of polysorbate 80. Importantly, MNCs fabricated from LMNPs showed the strongest darkening effect. From these results, in our system, we determined that MNCs fabricated from LMNPs using 50 mg polysorbate 80 exhibited good solubility and provided the greatest enhancement of MRI sensitivity.

**Figure 4:**
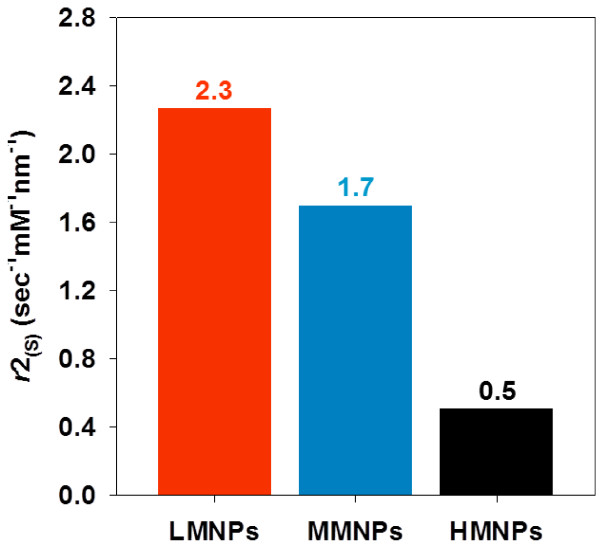
**The *r*2**_**(S) **_**(*r*2 enhancement divided by size increase of MNCs) for each PMNP.**

To investigate the efficiency of the engineered MNCs prepared by double-ligand modulation, we defined another form of relaxivity (*r*2_(S)_) that referred the *r*2 enhancement property based on size increase of MNCs. The *r*2 enhancement for each PMNP (107.8 ~ 68.5 s^−1^ mM^−1^ for LMNPs, 102.7 ~ 19.2 s^−1^ mM^−1^ for MMNPs, 44.3 ~ 19.3 s^−1^ mM^−1^ for HMNPs) were divided by size increase (59.9 ~ 42.6 nm for LMNPs, 65.1 ~ 15.8 nm for MMNPs, 66.6 ~ 17.1 nm for HMNPs). The *r*2_(S)_ values thus obtained were 2.3, 1.7, and 0.5 s^−1^ mM^−1^ nm^−1^ for LMNPs, MMNPs, and HMNPs, respectively (Figure 4). The positive value of *r*2_(S)_ indicated that MNC *r*2 enhancement was related to MNC size increase in association with using decreasing polysorbate 80 concentrations as the secondary-ligand modulation. However, the difference in *r*2_(S)_ among LMNPs, MMNPs, and HMNPs meant that the efficiency of the *r*2 enhancement through the engineering of MNCs depended on the primary-ligand modulation. Precisely, LMNPs possessed the highest *r*2_(S)_ (2.3 s^−1^ mM^−1^ nm^−1^), indicating that LMNPs were the most effective for creating MNCs with enhanced *r*2 values. Taken together, these results defined the precise primary and secondary ligand concentrations that work together to produce MNCs that are of optimal size and magnetic content for enhancing MRI *r*2 values.

## Conclusions

We successfully engineered MNCs based on double-ligand modulation to act as contrast agents and significantly enhance MRI sensitivity. The functions of primary and secondary ligands during MNC synthesis could be independently controlled by stepwise modulation processes. The density of individual MNPs in the MNCs was increased by decreasing the amount of oleic acid on the MNPs (primary-ligand modulation), and MNC size was increased by reducing the concentration of polysorbate 80 (secondary-ligand modulation). Together, these two effects effectively increase MNC *r*2 values. Our new MNC fabrication strategy using double-ligand modulation overcomes the limitation of MNC generation by single-ligand modulation alone and allows the precise regulation of MNC size, density, and magnetic properties to optimally enhance MRI. Moreover, our investigation provided a versatile and powerful model to engineer various secondary structures of diverse nanocrystals and to subsequently evaluate their physical properties.

## Competing interests

The authors declare that they have no competing interests.

## Authors’ contributions

BK carried out the ligand modulation and nanoemulsion and drafted the manuscript. JY conceived of the experimental design and condition. E-KL carried out the synthesis of magnetic nanoparticles. JP conceived of the particle relaxivity analysis. J-SS participated in the modification of magnetic resonance imaging sequence. HSP performed the statistical analysis. Y-MH and SH participated in the design of the study and drafted the manuscript. All authors read and approved the final manuscript.

## Supplementary Material

Additional file 1 **Figures S1 to S5 and Tables S1 to S3.** Figure S1. (**a**) X-ray diffraction pattern and (**b**) magnetic hysteresis curve of MNPs. S2. Derivative weight curve of pure oleic acid. S3. FT-IR spectra of pure oleic acid and MNPs (detailed analysis is presented in Table S1). S4. (**a**) Derivative weight curve of Fe-oleate precursor, (**b**) illustration for the interactions of oleic acid in Fe-oleate precursor. S5. Representative images of MNCs solution in the cubic cell according to the time of 0 (immediately), 6 and 24 hours. Table S1. FT-IR analysis of Figure S3. S2. Infrared frequencies and band assignments for the iron-carboxylate complexes. S3. Detailed values for the size and *r*2 of MNCs presented in Figure 3a, b. Click here for file
