# Supplementary material for: Double-ligand modulation for engineering magnetic nanoclusters
Source: Nanoscale Res Lett. 2013 Feb 22;8(1):104. doi: 10.1186/1556-276X-8-104 (PMC3614429; doi:10.1186/1556-276X-8-104)
Supplement: Additional file 1 — Figures S1 to S5 and Tables S1 to S3. Figure S1. (a) X-ray diffraction pattern and (b) magnetic hysteresis curve of MNPs. S2. Derivative weight curve of pure oleic acid. S3. FT-IR spectra of pure oleic acid and MNPs (detailed analysis is presented in Table S1). S4. (a) Derivative weight curve of Fe-oleate precursor, (b) illustration for the interactions of oleic acid in Fe-oleate precursor. S5. Representative images of MNCs solution in the cubic cell according to the time of 0 (immediately), 6 and 24 hours. Table S1. FT-IR analysis of Figure S3. S2. Infrared frequencies and band assignments for the iron-carboxylate complexes. S3. Detailed values for the size and r2 of MNCs presented in Figure 3a, b. [file 1556-276X-8-104-S1.doc]

**Supporting Information**

**“Double-Ligand Modulation for Engineering Magnetic Nanoclusters”**


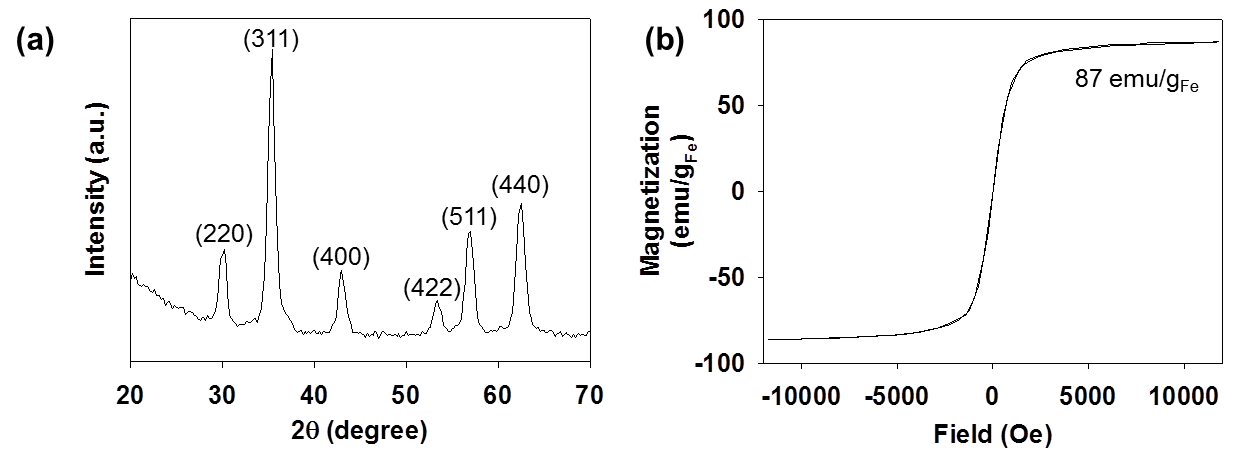


**Figure S1.** (a) X-ray diffraction pattern and (b) magnetic hysteresis curve of MNPs.


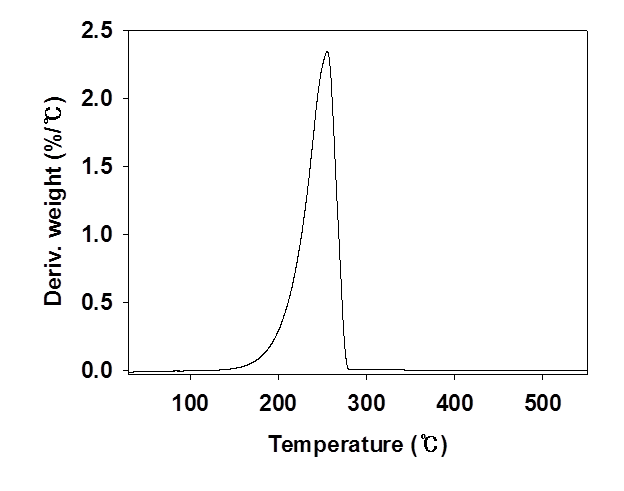


**Figure S2.** Derivative weight curve of pure oleic acid.


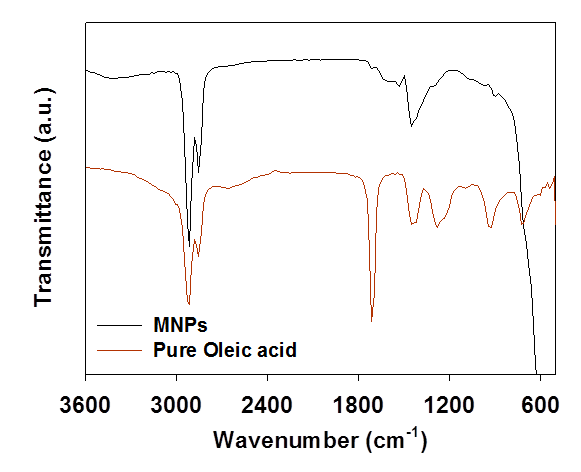


**Figure S3.** FT-IR spectra of pure oleic acid and MNPs (detailed analysis is presented in **Table S1**).


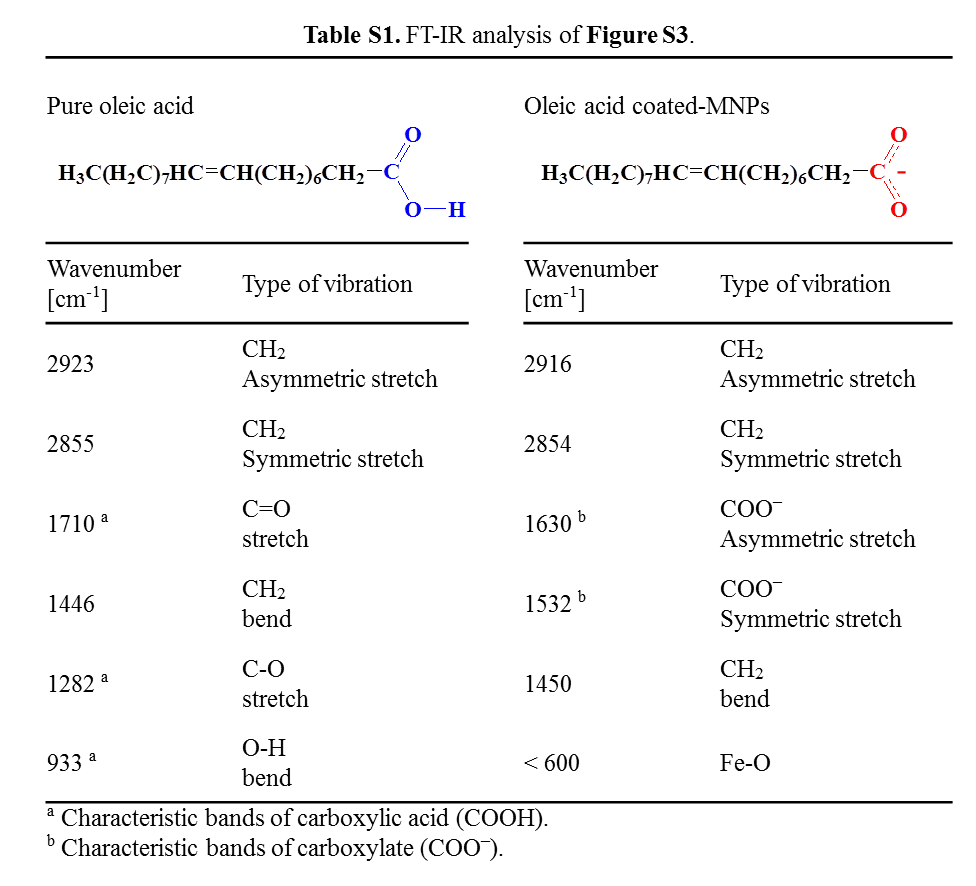


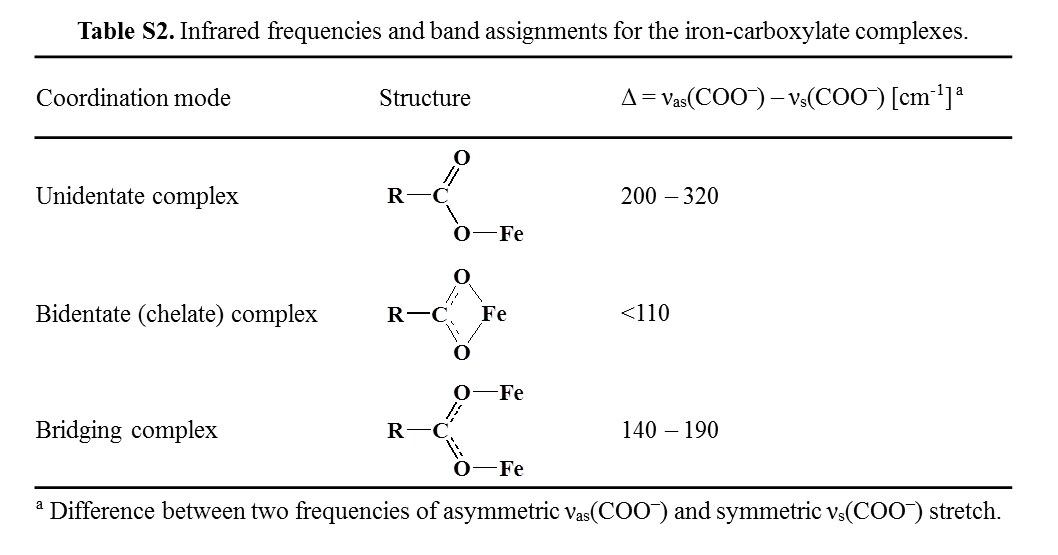


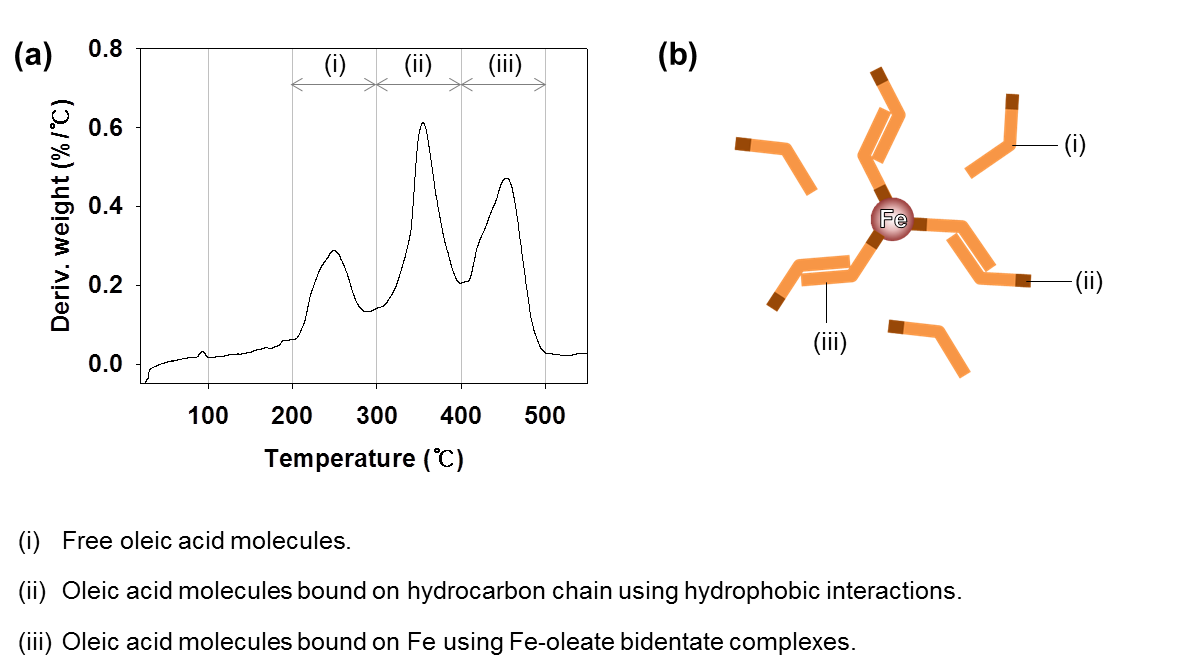


**Figure S4.** (a) Derivative weight curve of Fe-oleate precursor, (b) illustration for the interactions of oleic acid in Fe-oleate precursor


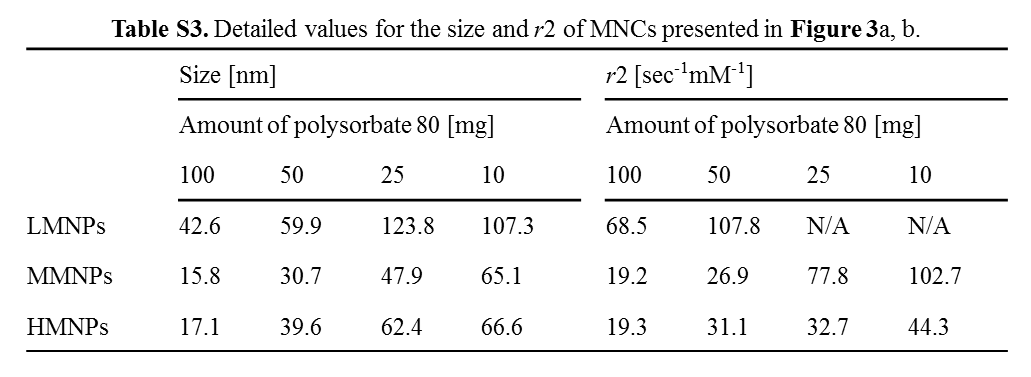


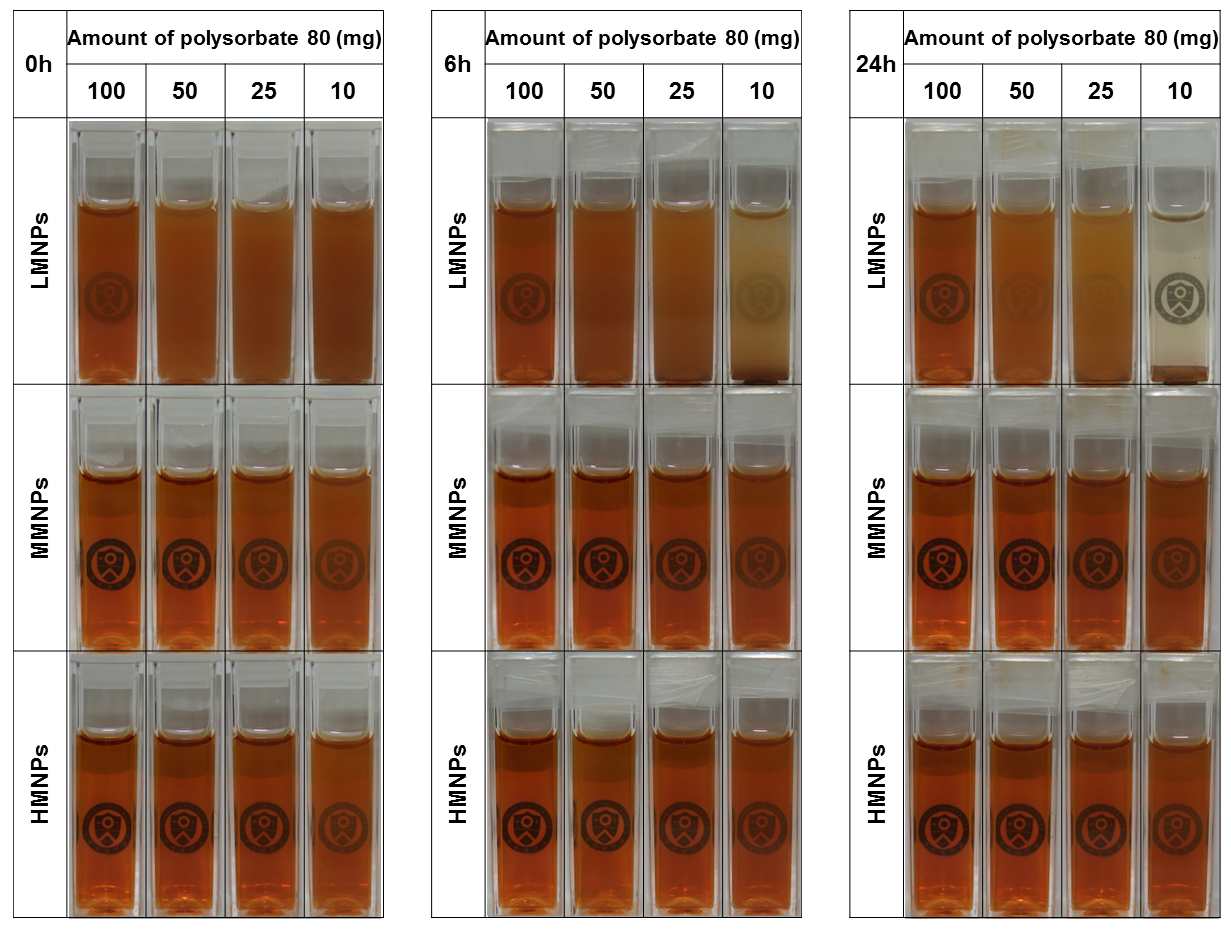


**Figure S5.** Representative images of MNCs solution in the cubic cell according to the time of 0 (immediately), 6 and 24 hours.
